# Motivations and strategies underlying the adoption of the front-of-pack labelling scheme Nutri-Score

**DOI:** 10.1093/eurpub/ckac130.099

**Published:** 2022-10-25

**Authors:** B Sarda, M Fialon, P Ducrot, AJ Serry, P Galan, E Kesse-Guyot, S Hercberg, M Touvier, C Julia

**Affiliations:** 1 EREN, Sorbonne Paris Nord University, Bobigny, France; 2 Public Health Department, Hopital Avicenne, Bobigny, France; 3 Physical Activity and Diet Department, Public Health France, Saint-Maurice, France

## Abstract

Front-of-pack nutrition labellings (FoPL) have gained traction in the European Union as tools to tackle the burden of nutrition-related chronic diseases. Multiple FoPL have been implemented by both governments and private actors. Under the European food labelling regulation, any FoPL is optional to display, although the legal framework is being revised for a harmonized FoPL to emerge. Since the implementation of the Nutri-Score in France in 2017, little data is available to understand the motivations and strategies underlying the adoption of the FoPL from a food business’ perspective. A cross-sectional study was conducted in March 2021 on food businesses having adopted the Nutri-Score in France through an online questionnaire. In total, 121 businesses completed the questionnaire, representing 32% of companies adopting the scheme and covering a variety of company types. Engaged businesses had a rather healthier portfolio of products according to the Nutri-Score (on average, 69% of engaged products were A or B and 12% were D or E), with disparities between retailers and national brands. Businesses mostly reported their engagement was motivated by a will to be transparent (76%) and to simplify nutritional information (67%) but still 19% reported that they engaged following external pressures. External pressures were more likely to play a role in the engagement if the company engaged in 2020 compared to those engaged before 2018 (P = 0.032), if the company was larger (P = 0.044) and if the scope of engagement included D or E products (poorer nutritional quality) (P = 0.033). Our study showed that the Nutri-Score, as an optional measure, is mainly used by businesses, especially national brands, as a tool to promote rather healthier products, while companies with products of lower nutritional quality are more likely to engage following external pressure. This highlights the relevance of enforcing a mandatory FoPL system, in line with the Farm to Fork strategy.

**Key messages:**

- Nutri-Score led to a selective use of the measure by food businesses, especially national brands, to promote rather healthier products.
- The relatively low engagement of companies with low nutritional value products highlights the relevance of enforcing a mandatory FoPL system, in line with the Farm to Fork strategy.

